# Synthesis and Pharmacological Activities of Some New Triazolo- and Tetrazolopyrimidine Derivatives

**DOI:** 10.3390/molecules181215051

**Published:** 2013-12-06

**Authors:** Saleh A. Bahashwan, Ahmed A. Fayed, Abd El-Galil E. Amr, Eman M. Flefel, Atef Kalmouch

**Affiliations:** 1 Pharmacology and Toxicology Department, College of Pharmacy, Taibah University, Al-Madinah Al-Munawara 3893, Saudi Arabia; 2 Respiratory Department (Applied Organic & Biochemistry Division), College of Medical Rehabilitation Sciences, Taibah University, Al-Madinah Al-Munawara 3893, Saudi Arabia; 3 National Research Center, Dokki, Cairo, Dokki 12622, Egypt; 4 Pharmaceutical Chemistry Department, Drug Exploration & Development Chair (DEDC), College of Pharmacy, King Saud University, Riyadh 11451, Saudi Arabia; 5 Deanship of Academic Services Preparatory Years, Health Science Track, Taibah University, Al Madinah Al-Monawarah 344, Saudi Arabia

**Keywords:** pyrazolopyrimidines, thienopyrimidines, triazolo- and tetrazolopyrimidines, anti-inflammatory and ulcerogenic activities

## Abstract

A new series of fused triazolo- and tetrazolopyrimidine derivatives **2**–**14** were synthesized and their anti-inflammatory and ulcerogenic activities were evaluated. The pharmacological screening showed that many of these obtained compounds have good anti-inflammatory activities, comparable to the reference drug. The toxicity of the compounds was also assayed via the determination of their LD_50_ values. The structures of newly synthesized compounds were confirmed by IR, ^1^H-NMR, MS spectral data and elemental analysis.

## 1. Introduction

In our previous work, some polycyclic heterocyclic derivatives were studied as 5α-reductase inhibitors, antiviral and anti-tumor agents [1]. Some of these compounds also showed aromatase and quinone reductase-2 inhibitors [2], anti-inflammatory, analgesic and antipyretic [3,4], and anti-arthritic and immunosuppressive activities [5]. Pyrimidines and fused pyrimidines, being integral parts of DNA and RNA, play an essential role in several biological processes and also have considerable chemical and pharmacological importance as antibiotics, antibacterials, cardiovascular as well as agrochemical and veterinary products [6,7,8,9,10,11,12]. Heterocyclic compounds play an important role in designing new classes of structural entities of medicinal importance with potentially new mechanisms of action. These heterocyclic compounds are well known to possess diverse pharmacological properties, *viz*. antimicrobial, analgesic, anti-inflammatory, anticancer, anticonvulsant and antimalarial activities [13]. On the other hand, we have reported that some of our new substituted heterocyclic compounds exhibited antiparkinsonian [14], antitumor [15,16], and anti-inflammatory [17] activities. In addition, during the last few years, condensed thienopyrimidine derivatives have received considerable attention. Many of these derivatives were found to possess a variety of pronounced activities such as anti-inflammatory and analgesic [18,19,20,21], antimicrobial [22,23,24,25], anti-avian influenza virus (H5N1) [26], anti-herpes simplex virus type-1 (HSV-1) and hepatitis-A virus (HAV), serotonin 5-HT_6_ receptor antagonist [27], antiarrhythmic [28] agent properties. Pyrimidine derivatives have been previously reported as platelet aggregation inhibitors, antagonists, anti-conceptive and anti-parkinsonism [29,30,31,32] agents. Heterocyclic compounds have also exhibited anthelmintic, anti HIV activity and hypoglycemic activities [33]. In view of these observations and as continuation of our previous works on heterocyclic chemistry, we report herein the synthesis of some new heterocyclic containing pyrazolothienopyrimidine moieties and the study of their anti-inflammatory activities in comparison to indomethacin used as positive control.

## 2. Results and Discussion

### 2.1. Chemistry

**Scheme 1:**
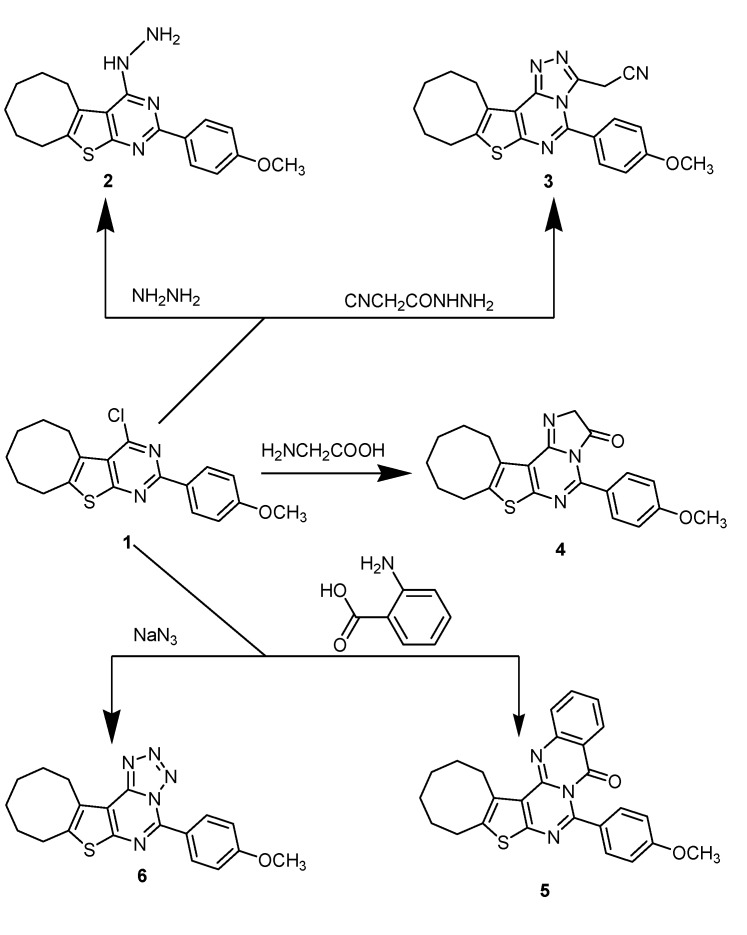
Synthetic route for compounds **2**–**6**.

**Scheme 2:**
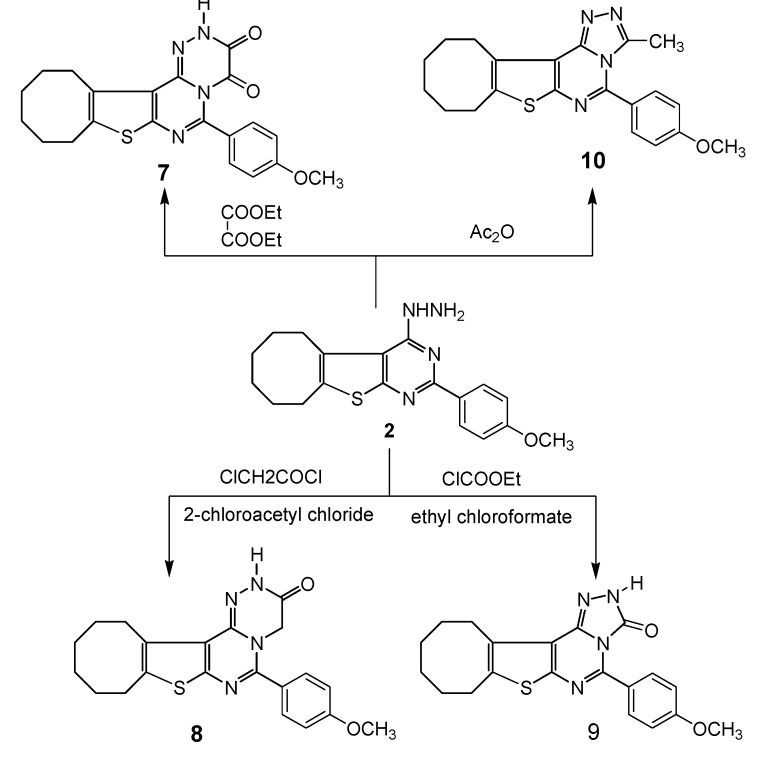
Synthetic route for compounds **7**–**10**.

2-(4-Methoxypheyl)-4-chlorocycloocteno[4,5]thieno[2,3-e]pyrimidine (**1**) [34] was treated with hydrazine hydrate to afford the corresponding hydrazinopyrimidine **2**. Reaction of compound **1** with cyanoacetylhydrazine in boiling butanol afforded the corresponding 3-(cyanomethyl)triazolo-pyrimidine **3**. Compound **1** was also reacted with amino acids to give imidazo derivatives **4** and **5**, respectively. Treatment of **1** with sodium azide in boiling acetic acid gave the corresponding tetrazolopyrimidine **6** (Scheme 1). On the other hand, when compound **2** was refluxed with diethyl oxalate or chloroacetylchloride it afforded triazino derivatives **7** and **8**, respectively. The IR and ^1^H-NMR spectra of **7** and **8** revealed the absence of the NH_2_ group. Also, reaction of compound **2** with ethyl chloroformate or acetic anhydride gave triazolopyrimidine derivatives **9** and **10**, respectively. The IR spectrum of compound **9** showed absorption bands due to C=O and NH; its ^1^H-NMR showed a singlet at 6.24 ppm due to the NH (amide) group, while the ^1^H-NMR of compound **10** showed a singlet at 2.16 ppm due to the CH_3_ group (Scheme 2).

**Scheme 3:**
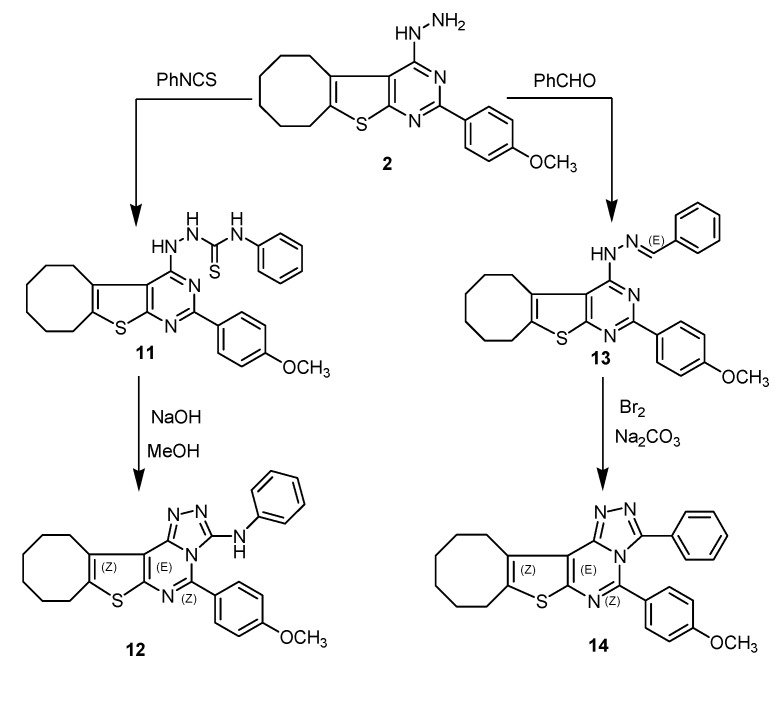
Synthetic route for compounds **11**–**14**.

Reaction of compound **2** with phenyl isothiocyanate in boiling ethanol yielded compound **11**, which was treated with methanolic sodium hydroxide to give the cyclized compound **12**. The ^1^H-NMR spectrum of compound **12** showed a singlet at δ = 10.23 ppm due to the NH group. Finally, compound **2** was reacted with benzaldehyde to give arylidenehydrazine derivative **13**, which was cyclized with bromine and sodium carbonate to give triazolopyrimldine **14** (Scheme 3).

### 2.2. Pharmacological Screening

Two pharmacological activities namely; anti-inflammatory and ulcerogenic activities were tested despite their different biological receptors. Eleven representative compounds—**2**–**10**, **12** and **14**—were evaluated as anti-inflammatory agents. Compounds **2** and **10** were also evaluated for ulcerogenic activity. The activities of these compounds are different according to the structure and function groups (Tables 1 and 2).

#### 2.2.1. Anti-inflammatory Activity

The anti-inflammatory results after 1 h (percent inhibition of edema obtained by the reference drug and tested compounds, respectively) show that compounds 2, **3**, **6**, **9** and **10** possess strong anti-inflammatory activity (42.3 ± 1.1, 37.2 ± 1.3, 34.5 ± 1.2, 31.2 ± 1.2 and 39.1 ± 1.5% of inhibition, respectively), in comparison to that of indomethacin (44.7 ± 1.2%). Compounds **7**, **8** and **12** possess moderate anti-inflammatory activity (20.8 ± 1.4, 21.3 ± 1.2 and 19.4 ± 1.6% of inhibition, respectively), in comparison to that of indomethacin (44.7 ± 1.2%). Compounds **4**, **5** and **14** possess weak anti-inflammatory activity from (16.5 ± 1.3, 11.7 ± 1.1 and 7.3 ± 1.1% of inhibition, respectively), in comparison to that of indomethacin (44.7 ± 1.2%). The anti-inflammatory assay results (percent inhibition of edema obtained by the reference drug and tested compounds) after 2 h show that compounds 2, **3**, **6**, **9** and **10** possess strong anti-inflammatory activity (49.3 ± 1.1, 46.3 ± 1.5, 42.1 ± 1.3, 35.1 ± 1.5 and 48.4 ± 1.2% of inhibition, respectively), in comparison to that of indomethacin (52.4 ± 1.2%). Compounds **7**, **8** and **12** possess moderate anti-inflammatory activity (22.3 ± 1.3, 24.6 ± 1.1 and 21.3 ± 1.3% of inhibition, respectively) in comparison to that of indomethacin (52.4 ± 1.2%). Compounds **4**, **5** and **14** possess weak anti-inflammatory activity from (17.2 ± 1.1, 13.2 ± 1.2 and 4.8 ± 1.2% of inhibition, respectively) in comparison to that of indomethacin (52.4 ± 1.2%). The results of anti-inflammatory after 3 h show that compounds 2, **3**, **6**, **9** and **10** possess strong anti-inflammatory activity (57.1 ± 1.4, 54.4 ± 1.1, 48.2 ± 1.2, 40.2 ± 1.6 and 55.6 ± 1.1% of inhibition, respectively) in comparison to that of indomethacin (61.2 ± 1.3%). Compounds **7**, **8** and **12** possess moderate anti-inflammatory activity (25.1 ± 1.4, 26.5 ± 1.2 and 23.3 ± 1.2% of inhibition, respectively) in comparison to that of indomethacin (61.2 ± 1.3%). Compounds **4**, **5** and **14** possess weak anti-inflammatory activity (from 19.3 ± 1.2, 15.2 ± 1.5 and 11.2 ± 1.5% of inhibition, respectively) in comparison to that of indomethacin (61.2 ± 1.3%) (Table 1).

**Table 1:** Anti-inflammatory activity of the synthesized compounds.

Compound No.	Edema inhibition (Means ± E.M) ^a,b^ (%)		
	1 h	2 h	3 h
2	42.3 ± 1.1	49.2 ± 1.2	57.1 ± 1.4
3	37.2 ± 1.3	46.3 ± 1.5	54.4 ± 1.1
4	16.5 ± 1.3	17.2 ± 1.1	19.3 ± 1.2
5	11.7 ± 1.1	13.2 ± 1.2	15.2 ± 1.5
6	34.5 ± 1.2	42.1 ± 1.3	48.2 ± 1.2
7	20.8 ± 1.4	22.3 ± 1.3	25.1 ± 1.4
8	21.3 ± 1.2	24.6 ± 1.1	26.5 ± 1.2
9	31.2 ± 1.2	35.1 ± 1.5	40.2 ± 1.6
10	39.1 ± 1.5	48.4 ± 1.2	55.6 ± 1.1
12	19.4 ± 1.6	21.3 ± 1.3	23.3 ± 1.2
14	7.3 ± 1.1	9.8 ± 1.2	11.2 ± 1.5
Indomethacin	44.7 ± 1.2	52.4 ± 1.2	61.2 ± 1.3

^a^ Dose 5 mg/kg b.m(p.o.); ^b^ n = 6.

#### 2.2.2. Ulcerogenicity

Compound**s** **2** and **10** showed higher anti-inflammatory activities than the other compounds so they were also screened for their ulcerogenic activity at dose levels of 10, 50 and 100 mg/kg b.m. (Table 2). The tested compound **2** and **10** showed no ulcerogenic activity of 1.4 to 2.1 mm.

**Table 2:** Ulcerogenic activities of compounds **2** and **10** (Gastric Ulceration in mice ^a^).

Compd. No.	Dose (mg/kg)^a^		
	10	50	100
Control	0/6	0/6	0/6
2	0/6(0)	0/6(0)	0/6(0)
10	0/6(0)	0/6(0)	0/6(0)
Indomethacin	3/6(1.4 ± 0.2) ^b^^,c^	5/6(1.9 ± 0.2) ^b^^,c^	6/6(2.1 ± 0.2) ^b^^,c^

^a^ number of mice lesions bigger than 0.5 mm in length per total no. of mice. ^b^ mean ulcer lesions ± SEM (mm) (n = 6) in parentheses. ^c^ significant difference at *p* ≤ 0.05 compared to the control.

#### 2.2.3. Acute Toxicity

To determine the acute toxicity of the compounds, the LD_50_ values for compounds **2**, **3**, **6**, **9** and **10** were determined by injecting different gradual increased doses of the tested compounds into male adult mice, then calculating the dose corresponding to 50% animal death (Table 3).

**Table 3:** Acute toxicity (LD_50_) of some of synthesized compounds.

Compound No.	LD_50_[mg/kg] *
2	2.514 ± 0.012
3	2.131 ± 0.013
6	2.047 ± 0.012
9	1.832 ± 0.012
10	2.341 ± 0.011
Indomethacin	1.817 ± 0.015

* Significant difference at *p* ≤ 0.05 compared to the control.

### 2.3. Structure Activity Relationships

Structure activity relationships based on the obtained results indicated that substitution of pyrimidine derivatives modulated the anti-inflammatory activity. Compounds **2**, **3**, **6**, **9** and **10** have high anti-inflammatory activity due to the presence of electron-donating moieties which increase the pharmacological activity. The sequence of anti-inflammatory properties regarding substitution of electron–donating group in pyrimidine derivatives is as follows: hydrazine > methyl > cyanomethyl > tetrazine > amide as exhibited in compounds **2** > **10** > **3** > **6** > **9**, respectively. Compounds **7**, **8** and **12** have moderate anti-inflammatory activity due to the presence of electron-withdrawing moieties which decrease the pharmacological activity. The sequence of anti-inflammatory properties regarding substitution of electron-withdrawing groups in pyrimidine derivatives is: amide > carbonyl adjacent to amide > phenyl amine as exhibited in compounds **8** > **7** > **12**, respectively. Also, compounds **4**, **5** and **14** have weak anti-inflammatory activity due to the presence of electron-withdrawing moieties which strongly decrease the pharmacological activity. The sequence of anti-inflammatory properties regarding electron-withdrawing group substitution in pyrimidine derivatives is in this case: carbonyl of imidazole > carbonyl of pyrimidine > phenyl as exhibited in compounds **4** > **5** > **14**, respectively.

## 3. Experimental

### 3.1. General

Melting points were determined on open glass capillaries using an Electrothermal IA 9000 SERIES digital melting point apparatus (Electrothermal, Essex, UK) and are uncorrected. Elemental analyses were performed with all final compounds on an Elementar Vario EL, located at the Microanalytical Unit, National Research Centre, Cairo, Egypt and were found within *±* 0.4% of the theoretical values. The IR spectra (KBr) were recorded on a FT IR-8201 PC spectrophotometer. The ^1^H-NMR spectra were measured with a Jeol FTGNM-EX 270, 270 MHz instrument in DMSO-*d*_6_ and the chemical shifts were recorded in (δ, ppm) relative to TMS. The mass spectra were run at 70 eV with a Finnigan SSQ 7000 spectrometer using EI and the *m/z* values are indicated in Dalton. TLC (silica gel, aluminum sheets 60F_254_, Merck, Darmstadt, Germany) was used to follow the reactions.

### 3.2. Chemical Synthesis

*2-(4-Methoxyphenyl)-4-hydrazinocycloocteno[4.5]thieno[2.3-d]pyrimidine* (**2**). A mixture of **1** (3.5 g, 0.01 mol) and hydrazine hydrate (0.68 g, 0.01 mol) in *n-*butanol (20 mL) was heated under reflux for 6 h, the formed solid was filtered off and crystallized from ethanol to give **2** as a green powder (76%); mp 235–237 °C; IR (KBr, ν, cm^−1^): 3336–3420 (NH, NH_2_); ^1^H-NMR (DMSO-*d*_6_, δ, ppm): 1.28–1.93 (m, 8H, 4CH_2_), 2.74–3.12 (m, 4H, 2CH_2_), 3.52 (s, 3H, OCH_3_), 6.45 (s, 2H, NH_2_, D_2_O exchangeable), 7.14–7.56 (m, 4H, Ar-H), 9.12 (s, 1H, NH, D_2_O exchangeable); MS: *m/z* (%): 354 (M^+^, 31) and 216 (100, M^+^-C_7_H_10_N_2_O). Anal. calcd for C_19_H_22_N_4_OS (354.43): C, 64.40; H, 6.21; N, 15.81; Found: C, 64.13; H, 6.02; N, 15.59.

*5-(4-Methoxyphenyl)-3-cyanomethylcycloocteno[4.5]thieno[2.3-d]triazolo[1,2,4]-[4,5-a]pyrimidine* (**3**). A mixture of **1** (3.5 g ,0.01 mol) and cyanoacetyl hydrazine (0.99 g, 0.01 mol) in *n*-butanol (30 mL) was refluxed for 8 h, the product obtained after cooling was crystallized from pet. ether (b.p. 40–60 °C) to give **3** as a reddish-brown powder (72%); mp 289–291°C; IR (KBr, ν, cm^−1^): 2219 (CN); ^1^H-NMR (DMSO-*d*_6_, δ, ppm): 1.25–1.84 (m, 8H, 4CH_2_), 2.36–2.87 (m,4H, 2CH_2_), 3.18 (s, 3H, OCH_3_), 3.46 (s, 2H, CH_2_), 7.21–7.63 (m, 4H, Ar-H); MS: *m/z* (%): 403 (M^+^, 19) and 256 (100, M^+^-C_9_H_9_NO). Anal. calcd. for C_22_H_21_N_5_OS (403.45): C, 65.50; H, 5.21; N, 17.36; Found: C, 65.24; H, 5.01; N, 17.12.

#### Synthesis of Compounds **4** and **5**

Amino acids, namely glycine and anthranilic acid (0.01 mol), respectively, were poured into a solution of **1** (3.5 g, 0.01 mol) in ethanol (20 mL). The reaction mixture was refluxed for 5 h then, the solid separated was refluxed with acetic anhydride (15 mL) for 3 h and the product obtained after cooling was crystallized from a proper solvent to give **4** and **5**, respectively.

*5-(4-Methoxyphenyl)-3-oxo-cycloocteno[4.5]thino[2,3-d]-2H-pyrazolo[3,2-c]-pyrimidine* (**4**). From ethanol, a brown powder (59%); mp over 300 °C; IR (KBr, ν, cm^−1^): 1730 (C=O); ^1^H-NMR (DMSO-*d*_6_, δ, ppm): 1.36–1.78 (m, 8H, 4CH_2_), 2.23–2.75 (m, 4H, 2CH_2_), 2.91 (s, 2H, CH_2_, imidazole), 3.18 (s, 3H, OCH_3_) and 7.12–7.58 (m, 4H, Ar-H); MS: *m/z* (%): 379 (M^+^, 24) and 216 (100, M^+^-C_9_H_11_NO_2_). Anal. calcd. for C_21_H_21_N_3_O_2_S (379.43): C, 66.49; H, 5.54; N, 11.08; Found: C, 66.21; H, 5.19; N, 10.82.

*8-(4-Methoxyphenyl)-6-oxo-cycloocteno[4.5]thino[2,3-d]pyrimidino[4.3-b]quinazoline* (**5**). From methanol, a yellow powder (71%); mp over 250 °C; IR (KBr, ν, cm^−1^): 1732 (C=O); ^1^H-NMR (DMSO-*d*_6_, δ, ppm): 1.31–1.54 (m, 8H, 4CH_2_), 1.91–2.14(m, 4H, 2CH_2_ ), 2.91 (s, 3H, OCH_3_) and 7.16–7.64 (m, 8H, Ar-H); MS: *m/z* (%): 455 (M^+^, 25); 264 (100, M^+^-C_13_H_19_O). Anal. calcd. for C_26_H_23_N_4_O_2_S (455.51): C, 70.74; H, 5.21; N, 9.52; Found: C, 70.51; H, 5.01; N, 9.26.

*5-(4-Methoxyphenyl)-cycloocteno[4.5]thieno[2.3-d]tetrazolo[4.5-e]pyrimidine* (**6**). A solution of **1** (3.5 g, 0.01 mol) and sodium azide (0.65 g, 0.01 mol) in glacial acetic acid (30 mL) was refluxed for 3 h. The product obtained after cooling was crystallized from benzene to give **6** as a green powder (59%); mp 254–256 °C; IR (KBr, ν, cm^−1^): 1621–1435 (C=N, C=C), 1281 (N-N=N), 1136 (tetrazole ring); ^1^H-NMR (DMSO-d_6_, δ, ppm): 1.43–1.85 (m, 8H, 4CH_2_), 2.87–2.92 (m, 4H, 2CH_2_), 3.15 (s, 3H, OCH_3_) and 7.26–7.38 (m, 4H, Ar-H); MS: m/z (%) ; 365 (M^+^, 42) and 216 (100, M^+^-C_7_H_7_N_3_O). Anal. calcd. for C_19_H_19_N_5_OS(365.41): C, 62.46; H, 5.20; N, 19.17; Found: C, 62.14; H, 5.04; N, 19.03.

*6-(4-Methoxyphenyl)-3,4-dioxo-2H-cycloocteno[4.5]thieno[2,3-d]triazino[1,2.4]-[3,4-a]pyrimidine* (**7**). A solution of **2** (3.50 g, 0.01mol) and diethyl oxalate (1.46 g, 0.01 mol) in ethanol (30 mL) was refluxed for 8 h.; The solid obtained after cooling was filtered and crystallized from ethanol to give **7** as a reddish brown solid (64%); mp over 300 °C; IR (KBr, ν, cm^−1^): 3315 (NH), 1730 (C=O amide, C-3 of triazine), 1728 (C=O amide of C-4 triazine); ^1^H-NMR spectrum (DMSO-*d*_6_, δ, ppm): 1.32–1.91 (m, 8H, 4CH_2_), 2.67–2.82 (m, 4H, 2CH_2_), 3.25 (s, 3H, OCH_3_), 7.24–7.38 (m, 4H, Ar-H) and 10.17 (s, 1H, NH; D_2_O exchangeable); MS: *m/z* (%); 408 (M^+^, 23) and 337 (100, M^+^-C_2_HNO_2_). Anal. calcd. for C_21_H_20_N_4_O_3_S (408.43): C, 61.76; H, 4.90; N, 13.72; Found: C, 61.32; H, 4.68; N, 13.43.

*6-(4-Methoxyphenyl)-3-oxo-2,4-dihydrocycloocteno[4,5]thieno[2,3-d]triazino-[1,2,4][3,4-a]pyrimidine* (**8**). A mixture of **2** (3.50 g, 0.01 mol) and chloroacetyl chloride (1.13 g, 0.01 mol) in pyridine (20 mL) was refluxed for 10 h, then, poured on dil. HCl. The solid product formed was crystallized from ethanol to give **8** as a brown powder (73%); mp 284–286 °C; IR (KBr, ν, cm^−1^): 3315 (NH), 1725 (C=O); ^1^H-NMR (DMSO-*d*_6_, δ, ppm): 1.15–1.82 (m, 8H, 4CH_2_), 1.98–2.65 (m, 4H, 2CH_2_), 3.52 (s, 3H, OCH_3_), 3.81 (s, 2H, CH_2_), 7.21–7.59 (m, 4H, Ar-H) and 9.01 (bs, 1H, NH; D_2_O exchangeable); MS: *m/z* (%): 394 (M^+^, 31) and 230 (100, M^+^-C_9_H_10_ NO_2_). Anal. calcd. for C_21_H_22_N_4_O_2_S (394.45): C, 63.95; H, 5.58; N, 14.21; Found: C, 63.63; H, 5.32; N, 14.03.

*5-(4-Methoxy)phenyl-3-oxo-2H-cycloocteno[4,5]thieno[2,3-d][1,2,4]triazolo[4,5-a]pyrimidine* (**9**). A mixture of **2** (3.50 g, 0.01 mol) and ethyl chloroformate (1.08 gm, 0.01 mol) in pyridine (20 mL) was refluxed for 12 h then, poured on dil. HCl. The solid obtained was crystallized from methanol to give **9** as a green powder (54%); mp > 300 °C; IR (KBr, ν, cm^−1^): 3320 (NH), 1731 (C=O); ^1^H-NMR (DMSO-*d*_6_, δ, ppm): 1.32–1.72 (m, 8H, 4CH_2_), 2.19–2.41 (m, 4H, 2CH_2_), 2.84 (s, 3H, OCH_3_), 6.24 (bs, 1H, NH; D_2_O exchangeable), 7.08–7.31 (m, 4H, Ar-H),; MS : *m/z* (%) ; 380 (M^+^, 19) and 216 (100, M^+^-C_8_H_8_N_2_O_2_). Anal. calcd. for C_20_H_20_N_4_O_2_S (380.42): C, 63.15; H, 5.26; N, 14.73; Found: C, 62.89; H, 5.04; N, 14.49.

*5-(4-Methoxyphenyl)-3-methyl-2H-cycloocteno[4,5]thieno[2,3-d][1,2,4]triazolo-[4,5-a] pyrimidine* (**10**) A solution of **2** (3.50 g, 0.01 mol) and acetic anhydride (20 mL) was heated under reflux for 2 h. The solid obtained after cooling was crystallized from pet. ether to give **10** as a yellow powder (54%); mp 272–274 °C; IR (KBr, ν, cm^−1^): 1418–1432 (C=N, C=C_Ar_); ^1^H-NMR (DMSO-*d*_6_, δ, ppm): 1.17–1.68 (m, 8H, 4CH_2_),1.93–2.81(m, 4H, 2CH_2_), 2.16 (s,3H,CH_3_), 2.21(s, 3H,OCH_3_) and 7.22–7.38 (m, 4H, Ar-H); MS: *m/z* (%); 379 (M^+^, 56) and 231 (100, M^+^-C_9_H_10_NO). Anal. calcd. for C_21_ H_22_N_4_OS (379.49): C, 66.67; H, 5.82; N, 14.81; Found: C, 66.39; H, 5.57; N, 14.53.

*N-[2-(4-Methoxyphenyl)cycloocteno[4,5]thieno[2,3-d]pyrimidino-4-yl]-N-phenylthio-semicarbazide* (**11**). A mixture of **2** (3.50 g, 0.01 mol) and phenyl isothiocyanate (2.50 mL) in ethanol (30 mL) was refluxed for 5 h, the product formed after cooling was collected and crystallized from methanol to give **11** as a reddish-brown solid (68%); mp 189–191 °C; IR (KBr, ν, cm^−1^): 3,342–3,415 (3 NH), 1,310 (C=S); ^1^H-NMR (DMSO-*d*_6_, δ, ppm): 1.26–1.91 (m, 8H, 4CH_2_), 2.05–3.18 (m, 4H, 2CH_2_), 3.42 (s, 3H, OCH_3_), 7.21–7.46 (m, 9H, Ar-H), 10.22 (s, 1H, NH exchangeable with D_2_O), 10.54 (s, 1H, NH; D_2_O exchangeable) and 11.15 (s, 1H, NH, D_2_O exchangeable); MS: *m/z* (%); 489 (M^+^, 31) and 216 (100, M^+^-C_14_ H_15_N_3_OS). Anal. calcd. for C_26_ H_27_N_5_OS_2_ (489.59); C, 63.80; H, 5.52; N, 14.31; Found: C, 63.61; H, 5.25; N, 14.19.

*5-(4-Methoxyphenyl)-3-anilinocycloocteno[4,5]thieno[2,3-d[1,2,4]triazolo[4,5-c]pyrimidine* (**12**) A mixture of **11** (4.89 g, 0.01 mol) and sod.hydroxide (0.40 g, 0.01 mol) in methanol (20 mL) was refluxed for 4 h. The product formed after cooling was collected and crystallized from acetic acid to give **12** as white powder (69%); mp 257–259 °C; IR (KBr, ν, cm^−1^): 3316–3350 (NH; NH_2_); ^1^H-NMR (DMSO-*d*_6_, δ, ppm): 1.32–1.74 (m, 8H, 4CH_2_), 1.89–2.61 (m, 4H, 2CH_2_), 2.95 (s, 3H, OCH_3_), 7.31–7.75 (m, 9H, Ar-H) and 10.23 (bs, 1H, NH, D_2_O exchangeable); MS: *m/z* (%); 455 (M^+^, 21) and 228 (100, M^+^-C_13_H_13_N_3_O). Anal. calcd for C_26_H_25_N_5_OS (455.52): C, 68.57; H, 5.49; N, 15.38; Found: C, 68.21; H, 5.16; N, 15.14.

*2-(4-Methoxyphenyl)-4-(N-benzylidenehydrazino)cycloocteno[4,5]thieno2,3-d]pyrimidine* (**13**) A mixture of **2** (3.50 g, 0.01 mol) and benzaldehyde (2.50 mL) in ethanol (20 mL) was heated under reflux for 2 h then cooled, The separated solid was filtered, dried and crystallized from pet. ether (b.p. 40–60 °C) to afford **13** as a green powder (48%); mp 174–176 °C; IR (KBr, ν, cm^−1^): 3350 (NH); ^1^H-NMR (DMSO-*d*_6_, δ, ppm): 1.25–1.82 (m, 8H, 4CH_2_), 3.06–3.14 ( m, 4H, 2CH_2_), 3.32 (s, 3H, OCH_3_) ,3.65 (s, 1H, CH=N), 7.21–7.78 (m, 9H, Ar-H) and 10.42 (bs, 1H, NH ;D_2_O exchangeable); MS: *m/z* (%); 442 (M^+^, 27) and 323 (100, M^+^-C_7_H_7_N_2_). Anal. calcd. for C_26_H_26_N_4_OS (442.51): C, 70.58; H, 5.88; N, 12.67; Found: C, 70.31; H,5.64; N, 12.39.

*5-(4-Methoxyphenyl)-4-phenylcycloocteno[4,5]thieno[2,3-d][1,2,4]triazolo[4,5-c]pyrimidine* (**14**) To a stirred mixture of **13** (4.42 g, 0.01 mol) and anhydrous sodium bicarbonate (1.68 g, 0.02 mol) in chloroform (20 mL) bromine (1.50 mL) was added. The mixture was stirred at room temperature for 5 h, then left to stand overnight, and evaporated under vacuum. The residue was triturated with ice-cold water, and the product formed was filtered, washed with water, dried and crystallized from ethanol to give **14** as a reddish brown solid (63%); mp over 300 °C; IR (KBr, ν, cm^−1^): 1620‒1435 (C=N_Ar_, C=C_Ar_); ^1^H-NMR (DMSO-*d*_6_, δ, ppm): 1.19–1.72 (m, 8H, 4CH_2_), 2.94–3.22 (m, 4H, 2CH_2_), 3.41 (s, 3H, OCH_3_) and 7.25–7.64 (m, 9H, Ar-H); MS: *m/z* (%); 440 (M^+^, 42) and 256 (100, M^+^-C_13_H_12_O). Anal. calcd. for C_26_H_24_N_4_OS (440.50); C, 70.91; H, 5.45; N, 12.72; Found: C, 70.65; H, 5.21; N, 12.42.

### 3.3. Pharmacological Screening

#### 3.3.1. Animals

Female albino mice (16–18 g) and Sprague Dawley mice (100 g) obtained from the Theodor Bilharz Research Institute (TBRI, Giza city, Egypt) were used. Approval of the institutional animal ethical committee for the animal studies was obtained from the Office of Environmental Health and Radiation Safety, ACUC Protocol 1096-5. The animals were maintained according to accepted standards of animal care.

#### 3.3.2. Anti-inflammatory Activity

Newly synthesized thienopyrimidine derivatives were dissolved in 0.5% carboxymethyl cellulose (CMC) as a homogeneous solution and administered intraperitonneally (i.p.). One hundred and eight rats were divided into eighteen groups, each group consisting of six animals. Anti-inflammatory activity of the compounds was studied in mice using carrageenan induced edema. A suspension of the tested compound and the reference drug, indomethacin in aqueous solution was administered orally at a dose 5 mg/kg. Control animals were treated with 0.5% CMC only. After 30 min, 0.1 mL of freshly prepared 1.0% carrageenan solution (in formol saline) was injected into the sub-plantar region of the right hind paw according to Hernandez-Perez [35]. The right paw volume was measured using a digital plethysmometer (Model 7150, Ugo Basile, Varese, Italy), directly before and after 1, 2, 3 h, intervals after administration of the tested compounds.

#### 3.3.3. Ulcerogenic Activity

Seventy-two mice were divided into twelve groups. Ulcerogenic activity was evaluated after oral administration of the tested compounds or indomethacin at doses of 10, 50, and 100 mg/kg. Control mice received 0.5% CMC. Food but not water was removed 24 h before administration of the tested compounds. After 6 h, the mice were sacrificed; the stomach was removed and opened along the greater curvature, washed with distilled water and cleaned gently by dipping in saline. The mucosa damage for each stomach was examined using a stereoscopic microscope and compared with the reference drug indomethacin according to the reported procedure [36].

#### 3.3.4. Acute Toxicity

The median lethal doses (LD_50_) of the most active compounds **2**, **3**, **6**, **9** and **10** were determined in mice [37]. Groups of male adult mice, each of six animals, were injected i.p. with graded doses of each of the test compounds. The percentage of mortality in each group of animals was determined 24 h, after injection. Computation of LD_50_ was processed by a graphical method.

#### 3.3.5. Statistical Analysis

Assay results are shown as mean ± SE. Statistical analyses were carried out with Sigma Plot software (SPSS Inc., Chicago, IL, USA). One-way analysis of variance (ANOVA) followed by Tukey’s post test was used to assess the presence of significant differences. Differences were considered statistically significant at *p* ≤ 0.05.

## 4. Conclusions

The objective of the present study was to synthesize and investigate the anti-inflammatory activities of some new thienopyrimidine derivatives. The starting material **1** was used to synthesize the following compounds: hydrazine thinopyrimidine **2**, triazolothinopyrimidine **3**, benzopyrimidinothieno pyrimidine **5** and tetrazolothienopyrimidine **6**. Compound **2** was used to synthesize the triazino- and triazolothinopyrimidine derivatives **7**, **8**, **9** and **10**, respectively. Also, compound **2** was carried out to synthesize of triazolothinopyrimidine derivatives **12** and **14**. The newly synthesized compounds **2**–**10**, **12** and **14** were screened for their anti-inflammatory activity compared to indomethacin which was used as reference drug. Compounds **3**, **6**, **9** and **10** possess strong anti-inflammatory activity, and compounds **8**, **7** and **12** possess moderate anti-inflammatory activity, while compounds **4**, **5** and **14** possess weak anti-inflammatory activity.
